# Global research funding for traditional, complementary and integrative medicine

**DOI:** 10.2471/BLT.25.293527

**Published:** 2025-10-13

**Authors:** Amie Steel, Kirsten Baker, Jon Adams, Hope Foley, Tristan Carter, Sarah Charnaud, Gustavo Martin Rossell De Almeida, Philippe Doo-Kingue, Pradeep Dua, Pawankumar Ramesh Godatwar, Eunkyung Han, Geraldine Hill, Ossy Muganga Julius Kasilo, Immaculee Mukankubito, Adi Al-Nuseirat, Dorina Pirgari, Anna Laura Ross, João Paulo Souza, Robert F Terry, Geetha Krishnan Gopalakrishna Pillai

**Affiliations:** a Australian Research Consortium in Complementary and Integrative Medicine (ARCCIM), School of Public Health, Faculty of Health, University of Technology Sydney, Level 8, Building 10, 235–253 Jones St, Ultimo, NSW, 2006, Australia.; b Science Division, World Health Organization, Geneva, Switzerland.; c Pan American Health Organization, Washington, DC, United States of America.; d Traditional, Complementary and Integrative Healthcare (TCIH) Coalition.; e WHO Global Traditional Medicine Centre, World Health Organization, Geneva, Switzerland.; f WHO Regional Office for South-East Asia, New Delhi, India.; g WHO Regional Office for the Western Pacific, Manila, Philippines.; h WHO Regional Office for the Eastern Mediterranean, Cairo, Egypt.; i WHO Regional Office for Europe, Copenhagen, Denmark.; j BIREME, Evidence and Intelligence for Action in Health, Pan American Health Organization, São Paulo, Brazil.; k Special Programme for Research and Training in Tropical Diseases, World Health Organization, Geneva, Switzerland.; l Global Traditional Medicine Centre, World Health Organization, Jamnagar, India.

## Abstract

**Objective:**

To explore the global research funding landscape for traditional, complementary and integrative medicine.

**Methods:**

We conducted a three-part study to assess the global research funding landscape. First, we searched the Dimensions database and online sources using Microsoft Copilot and Google between 12 November 2024 and 22 January 2025 for relevant grants. Second, we analysed national research infrastructure using World Health Organization (WHO) data, verified by regional contacts (14 January–28 February 2025). Third, we appraised selected funders across WHO regions, evaluating funding schemes for innovation, capacity-building and alignment with traditional medicine paradigms.

**Findings:**

We identified 39 927 grants in the Dimensions database, with funding data available for 27 019 grants totalling 24.5 billion United States dollars (US$) for the years 1960 to 2024. Most grants (42.6%; 11 548) were valued under US$ 100 000, and half had a duration of 2–4 years. Cancer and cardiovascular diseases accounted for over half (8385/15 273) of topic-categorized grants, receiving US$ 5.8 billion and US$ 2.2 billion, respectively. Funders were concentrated in the Region of the Americas, and European and Western Pacific Regions. Only seven countries had schemes explicitly funding research for traditional, complementary and integrative medicine. Case study analysis of 40 schemes across 12 countries revealed limited support for traditional medicine paradigms, with few schemes meeting criteria for innovation, capacity-building or sensitivity to traditional knowledge systems.

**Conclusion:**

Funding for traditional medicine research remains disproportionately low relative to its global use. Strengthening support from research funding agencies is essential to achieving the goals of the WHO *Global traditional medicine strategy 2025–2034*.

## Résumé

**Objectif:**

Explorer le paysage mondial du financement de la recherche en médecine traditionnelle, complémentaire et intégrative.

**Méthodes:**

Nous avons mené une étude en trois parties afin d’évaluer le paysage mondial du financement de la recherche. Tout d’abord, nous avons recherché des subventions pertinentes dans la base de données Dimensions et d’autres sources en ligne à l’aide de Microsoft Copilot et de Google du 12 novembre 2024 au 22 janvier 2025. Ensuite, nous avons analysé les infrastructures nationales de recherche à l’aide des données de l’Organisation mondiale de la Santé (OMS), vérifiées par des contacts régionaux (du 14 janvier au 28 février 2025). Enfin, nous avons évalué certains bailleurs de fonds dans les régions de l’OMS, en analysant les programmes de financement destinés à l’innovation, au renforcement des capacités et à l’alignement sur les paradigmes de la médecine traditionnelle.

**Résultats:**

Nous avons identifié 39 927 subventions dans la base de données Dimensions, avec des données de financement disponibles pour 270 198 subventions, totalisant 24,5 millions de dollars américains (USD) pour les années 1960 à 2024. La plupart des subventions (42,6%; 11 548) étaient d’une valeur inférieure à 100 000 USD, et la moitié d’entre elles avaient une durée de 2 à 4 ans. Le cancer et les maladies cardiovasculaires représentaient plus de la moitié (8 385/15 273) des subventions classées par thème, recevant respectivement 5,8 milliards et 2,2 milliards USD. Les bailleurs de fonds étaient concentrés dans la région des Amériques, ainsi que dans les régions Europe et Pacifique occidental. Seuls sept pays disposaient de programmes finançant explicitement la recherche en médecine traditionnelle, complémentaire et intégrative. L’analyse de 40 programmes dans 12 pays a révélé un soutien limité aux paradigmes de la médecine traditionnelle, peu de programmes répondant aux critères d’innovation, de renforcement des capacités ou de sensibilité aux systèmes de connaissances traditionnels.

**Conclusion:**

Le financement de la recherche en médecine traditionnelle reste disproportionnellement faible par rapport à son utilisation mondiale. Il est essentiel de renforcer le soutien des organismes de financement de la recherche pour atteindre les objectifs de la *Stratégie mondiale de l’OMS pour la médecine traditionnelle 2025–2034*.

## Resumen

**Objetivo:**

Explorar el panorama mundial de la financiación de la investigación en medicina tradicional, complementaria e integrativa.

**Métodos:**

Se realizó un estudio en tres fases para evaluar el panorama mundial de la financiación de la investigación. Primero, se buscaron subvenciones pertinentes en la base de datos Dimensions y en fuentes en línea mediante Microsoft Copilot y Google, entre el 12 de noviembre de 2024 y el 22 de enero de 2025. Segundo, se analizó la infraestructura nacional de investigación utilizando datos de la Organización Mundial de la Salud (OMS), verificados por contactos regionales (del 14 de enero al 28 de febrero de 2025). Tercero, se evaluaron organismos financiadores seleccionados en las regiones de la OMS, examinando sus programas de financiación en cuanto a innovación, desarrollo de capacidades y alineación con los paradigmas de la medicina tradicional.

**Resultados:**

Se identificaron 39 927 subvenciones en la base de datos Dimensions, con datos financieros disponibles para 270 198 subvenciones por un total de 24,5 millones de dólares estadounidenses (US$) correspondientes a 1960-2024. La mayoría de las subvenciones (42,6%; 11 548) tenían un valor inferior a US$ 100 000 y la mitad presentaban una duración de entre dos y cuatro años. El cáncer y las enfermedades cardiovasculares representaron más de la mitad (8385/15 273) de las subvenciones clasificadas por tema, recibiendo US$ 5800 millones y US$ 2200 millones, respectivamente. Los organismos financiadores se concentraron en la Región de las Américas y en las Regiones de Europa y del Pacífico Occidental. Solo siete países contaban con programas que financiaban explícitamente la investigación en medicina tradicional, complementaria e integrativa. El análisis de 40 programas en 12 países reveló un apoyo limitado a los paradigmas de la medicina tradicional, con pocos programas que cumplieran los criterios de innovación, desarrollo de capacidades o sensibilidad hacia los sistemas de conocimiento tradicional.

**Conclusión:**

La financiación de la investigación sobre medicina tradicional sigue siendo desproporcionadamente baja en relación con su uso mundial. Reforzar el apoyo de los organismos financiadores de investigación es esencial para alcanzar los objetivos de la *Estrategia mundial de la OMS sobre medicina tradicional 2025-2034*.

## ملخص

**الغرض:**

استكشاف المشهد العالمي لتمويل أبحاث الطب التقليدي والتكميلي والتكاملي.

**الطريقة:**

قمنا بإجراء  دراسة من ثلاثة أجزاء لتقييم المشهد العالمي لتمويل الأبحاث. أولًا، قمنا بالبحث في قاعدة البيانات Dimensions والمصادر عبر الإنترنت باستخدام Microsoft Copilot وGoogle بين 12 نوفمبر/تشرين ثاني 2024، و22 يناير/كانون ثاني 2025 عن المنح ذات الصلة. ثانيًا، قمنا بتحليل البنية التحتية الوطنية للأبحاث باستخدام بيانات منظمة الصحة العالمية (WHO)، التي تم التحقق منها من خلال جهات اتصال إقليمية (14 يناير/كانون ثاني إلى 28 فبراير/شباط 2025). ثالثًا، قمنا بتقييم جهات تمويل مختارة في مناطق منظمة الصحة العالمية، مع تقييم خطط التمويل للابتكار، وبناء القدرات، والتوافق مع نماذج الطب التقليدي.

**النتائج:**

قمنا بتحديد 39927 منحة في قاعدة بيانات Dimensions، مع توفر بيانات التمويل لـ 270198 منحة بإجمالي 24.5 مليون دولار أمريكي، للسنوات من 1960 إلى 2024. تم تقدير معظم المنح (%42.6؛ 11548) بأقل من 100000 دولار أمريكي، وكان نصفها لمدة تتراوح بين 2 و4 سنوات. شكلت أمراض السرطان وأمراض القلب والأوعية الدموية أكثر من نصف المنح المصنفة حسب الموضوع (8385/15273)، حيث تلقت 5.8 مليار دولار أمريكي، و2.2 مليار دولار أمريكي على التوالي. تركزت جهات التمويل في منطقة الأمريكتين، ومنطقة أوروبا وغرب المحيط الهادئ. لم يكن لدى سوى سبع دول مخططات تمول بشكل صريح أبحاث الطب التقليدي والتكميلي والتكاملي. كشف تحليل دراسة الحالة لـ 40 مخططًا في 12 دولة عن دعم محدود لنماذج الطب التقليدي، مع وجود عدد قليل من المخططات التي تلبي معايير الابتكار، أو بناء القدرات، أو الحساسية لأنظمة المعرفة التقليدية.

الاستنتاج لا يزال تمويل أبحاث الطب التقليدي منخفضًا بشكل غير متناسب مقارنةً باستخدامه العالمي. يُعد تعزيز الدعم من وكالات تمويل الأبحاث أمرًا أساسيًا لتحقيق أهداف الاستراتيجية العالمية لمنظمة الصحة العالمية للطب التقليدي 2025 إلى * 2034 *.

## 摘要

**目的:**

旨在探讨关于传统、补充和整合医学的全球研究的资助现状。

**方法:**

我们开展了一项由三个部分构成的研究，旨在评估全球研究的资助现状。第一，我们在 2024 年 11 月 12 日至 2025 年 1 月 22 日期间搜索了 Dimensions 数据库和在线资源（使用 Microsoft Copilot 和谷歌），以查找相关资助项目。第二，我们运用世卫组织 (WHO) 的数据分析了国家研究基础设施，且各区域联系人对此进行了核实（2025 年 1 月 14 日至 2 月 28 日期间）。第三，我们评估了世卫组织各个区域中已选定的资助机构，重点评估那些旨在推动创新、加强能力建设和提升与传统医学范式的契合度的资助计划。

**结果:**

我们在 Dimensions 数据库中找到了 39,927 个资助项目，并发现 1960 年至 2024 年期间总金额达 2,450 万美元 (US$) 的 270,198 个资助项目的相关资助数据可用。大多数资助项目（占 42.6%，11,548 个）的金额低于 10 万美元，且半数项目的持续时间为 2 至 4 年。在按主题分类的资助项目中，癌症和心血管疾病相关项目所占比例超过一半 (8,385/15,273)，资助金额分别为 58 亿美元和 22 亿美元。资助机构主要集中分布在美洲区域、欧洲区域和西太平洋区域。仅七个国家制定了明确表明其资助传统、补充和整合医学研究的相关计划。我们对 12 个国家的 40 份计划进行了案例研究分析，且分析结果表明，这些计划对传统医学范式的支持极为有限，仅极少数计划在创新、能力建设或对传统知识体系的敏感性方面符合相关标准。

**结论:**

与传统医学在全球范围内的使用率相比，在传统医学研究方面投入的资金仍然太少。加大研究资助机构的支持力度是实现“世卫组织 2025-2034 年全球传统医学战略”目标的重要前提**。**

## Резюме

**Цель:**

Изучить глобальную структуру и характер финансирования исследований в области традиционной, комплементарной и интегративной медицины.

**Методы:**

Было проведено исследование в три этапа для оценки глобального характера и структуры финансирования исследований. Во-первых, был проведен поиск соответствующих грантов в базе данных Dimensions и источниках в Интернете с использованием Microsoft Copilot и Google в период с 12 ноября 2024 г. по 22 января 2025 г. Во-вторых, была проанализирована национальная инфраструктура исследований по данным Всемирной организации здравоохранения (ВОЗ), которую верифицировали через региональных контактных лиц (с 14 января по 28 февраля 2025 г.). В-третьих, авторы дали оценку некоторым выбранным источникам финансирования в различных регионах ВОЗ, оценивая схемы финансирования по параметрам инноваций, развития возможностей и соответствия парадигмам традиционной медицины.** **

**Результаты:**

Авторы обнаружили в базе данных Dimensions 39 927 грантов. Для 270 198 грантов, предоставленных в период с 1960 по 2024 год были доступны данные по финансированию, при этом общая сумма за указанный период составила 24,5 миллиона долларов США (долл. США). Большая часть грантов (42,6%, 11 548) составляла сумму менее 100 000 долл. США, половина была рассчитана на срок 2–4 года. На исследования в области рака и сердечно-сосудистых заболеваний пришлось более половины (8385/15 273) тематически категоризованных грантов с выделением 5,8 и 2,2 миллиарда долл. США соответственно. Финансирующие лица и организации были сосредоточены в Американском, Европейском и Западно-Тихоокеанском регионах. Схемы, явным образом финансирующие исследования в сфере традиционной, комплементарной и интегративной медицины, имелись только в семи странах. Анализ конкретных случаев для 40 схем в 12 странах показал, что имеется лишь ограниченная поддержка парадигм традиционной медицины и только несколько из этих схем соответствуют критериям инноваций, создания возможностей или внимательного отношения к традиционным системам знаний.

**Вывод:**

Финансирование исследований в области традиционной медицины остается непропорционально низким относительно ее глобального использования. Укрепление поддержки со стороны агентств, занимающихся финансированием исследований, крайне важно для достижения целей *Глобальной стратегии ВОЗ в области традиционной медицины на 2025–2034 гг*.

## Introduction

Traditional, complementary and integrative medicine encompasses a wide range of practices, products, knowledge and medical systems that differ from the dominant biomedical paradigm and curriculum.^1^ Global demand for traditional, complementary and integrative medicine is substantial:^2^^,^^3^ of the 179 World Health Organization (WHO) Member States providing information for the *WHO global report on traditional and complementary medicine 2019*, 170 acknowledged use of traditional and complementary medicine among their populations.^1^ In low- and middle-income countries, demand is especially high where biomedical services may be unavailable or inaccessible, and where Indigenous medicines remain rooted in cultural practices.^4^^–^^10^

The vision of the WHO* Global traditional medicine strategy 2025–2034*^11^ is to create universal access to safe, effective and people-centred traditional, complementary and integrative medicine for the health and well-being of all; while its goal is to maximize its contribution to the highest attainable standard of health and well-being. Achieving this goal requires a robust evidence base, supported by more research that is comprehensive, inclusive and reflective of diverse epistemological approaches.^12^

Research funding is fundamental to a research ecosystem and encompasses diverse funding mechanisms and sources. These mechanisms and sources can include government-allocated block funding, that is, funding explicitly allocated as an ongoing budgetary item; and competitive grant schemes via government agencies, philanthropic organizations and commercial entities, with each mechanism and source offering unique contributions.^13^ To support WHO’s new traditional medicine strategy, we aimed to explore the global research funding landscape for traditional, complementary and integrative medicine.

## Methods

We conducted a three-part study to assess the global funding landscape for traditional, complementary and integrative medicine (hereafter referred to as traditional medicine). First, we searched online resources for information about research grants. Second, we examined national research infrastructure for traditional medicine. Finally, we did a case study analysis of prominent funders. Full details of the methods are provided in the online repository.^14^

### Grant identification

#### Search

To identify research funding, we searched the Dimensions database (Digital Science & Research Solutions Inc., London, England), which includes information about awarded grants from participating countries. We used Microsoft Copilot (Microsoft, Redmond, United States of America), an artificial intelligence assistant; and the Google search engine (Google, Mountain View, United States) to search other online sources and to check results.

Three authors, with the assistance of a research librarian, developed detailed search strategies by testing a structured preparatory search across each web-based platform to identify relevant source terms, keywords and, in the case of Copilot, additional research questions used to guide the formulation of the search. The full strategies for the three searches are available in the online repository.^14^

One author searched the Dimensions database between 12 November and 18 December 2024. To identify grants not recorded in the database, the author also searched online sources using Copilot. A second author verified the identified grants in Dimensions, by searching the database on 21 January 2025. For grants identified through Copilot but not indexed in Dimensions, a second author verified the data through independent Copilot and Google online searches, conducted on 22 January 2025. Discrepancies were discussed and, where needed, data were further verified through online searches. 

The searches yielded information about research grants through publications, grants, policies, data sets and related publication metrics. 

#### Eligibility criteria 

We included all grants indexed in and categorized as traditional medicine in Dimensions. Grants identified through Copilot and Google searches that reported funding for traditional, complementary and integrative medicine were also eligible. 

#### Data extraction

We downloaded identified grant information as CSV files and imported the data into Stata SE 18 (StataCorp LLC, College Station, United States) for analysis. We used the Stata duplicates command to detect duplicates. Identified duplicates were checked using the translated abstract title in Dimensions and the grant number provided by the funder. We only removed items if there was duplication of funding amount, abstract title and grant number within the same record.

For each WHO Member State, three authors extracted data from identified grants awarded for research, the traditional medicine specificity of such grants, awarding of philanthropic funding, the number and value of grants and associated publications. Of included grants, we analysed the grant abstracts to classify which traditional medicine topics were mentioned. Using the funding amounts provided by Dimensions at the time of the award, we used an online calculator^15^ to adjust for inflation and calculate the present-day value in United States dollars (US$), based on the consumer price index. To detect any discrepancies in the extracted data, one author checked the data tables. Any discrepancies were resolved through discussion between the two authors, with a third author mediating if necessary. 

As charitable and not-for-profit funding comprise an important and distinct component of health research funding, we checked the philanthropic status of individual funders through Copilot and Google searches.

### Infrastructure analysis

In alignment with the *WHO global report on traditional and complementary medicine 2019*,^1^ we examined four components of government infrastructure and its research: (i) government-funded national traditional medicine centres; (ii) government departments, ministries or agencies focused on traditional medicine; (iii) national traditional medicine policies or frameworks; and (iv) regulation of traditional medicine products or practice. We assessed the centres presented in the WHO report using Copilot and Google searches to identify each centre’s objectives, scope and funding mechanisms. To ensure the list was current and accurate, we used WHO regional office contacts, who verified and updated this list between 14 January and 28 February 2025. To assess the completeness of Dimensions data, we conducted online searches for countries with national centres responsible for administering government grant schemes.

### Case study funder analysis

We undertook a case study approach to critically appraise research funding schemes and assess their support for innovation, capacity-building and research that is sensitive to traditional medicine paradigms. This stage involved a content analysis of funding rules from selected grant schemes. We selected case study funders in three steps: (i) the most prominent funders identified in the Dimensions search for each WHO region, based on the number of grants awarded for traditional medicine; (ii) schemes identified using Google or Copilot as being specific to traditional, complementary and integrative medicine research including schemes not indexed in Dimensions; and (iii) for countries with specific schemes identified in Step 2, the top Dimensions-indexed funder was also included, even if the most prominent funder in that WHO region (from Step 1) was based in a different country. 

To ensure representativeness, we calculated the proportional contribution of each funder and their country to national and regional traditional medicine funding. For each funder, we reported the mean grant amount, grant duration, start date and total grant-linked publications using Dimensions data. We also assessed research impact of funders indexed in Dimensions, by calculating mean citations per funded publication as well as using Dimensions-linked Altmetrics (Clarivate, London, England) data.

We purposely selected up to five funding schemes per funder to illustrate the diversity in funding focus: (i) individuals; (ii) organizations; (iii) projects; (iv) topics specific to traditional medicine; and (v) commercial partnerships. Using a structured data extraction form, we extracted data for each scheme on funding amount, grant duration, methodological constraints, budget limitations, relevance and availability of funding rules. Further details are provided in the online repository.^14^

Two authors critically appraised funding rules using criteria from prior research and adapted from the Contemporary Implementation of Traditional knowledge and Evidence framework.^16^ Appraisal domains included innovation,^17^^,^^18^ capacity-building^19^ and sensitivity to traditional medicine-specific knowledge. Any disagreements during the appraisal were resolved through discussion with a third author. The appraisal domains and criteria are presented in Table 1 and in the online repository.^14^

**Table 1:** Criteria for critical appraisal domains

Appraisal category, level of assessment, criteria	Definition of appraisal category, level of assessment, criteria	Response option	No.
**Scheme level (*n* = 40)**			
Research innovation			
Longer funding duration	Funding with longer duration to support more complex and ground-breaking research projects	Yes (3 years or more)	16
		Partial (2 to 3 years)	7
		No (less than 2 years)	6
		NA	11
Scheme flexibility	Breadth of methods and topics accepted, with flexibility for researcher-driven question design	Yes	14
		Some	15
		No	7
		Unclear	4
Multidisciplinary	Preferences multidisciplinary work within teams	Yes	8
		Scope allows	18
		No	11
		Unclear	3
Research capacity-building			
Relationship to clinician	Clinician involvement in team or project	Yes	6
		Scope allows	6
		No	21
		Unclear	5
		NA	2
Relevance to practice	Practical and clinical relevance of topic or issue	Yes	6
		Scope allows	13
		No	14
		Unclear	5
		NA	2
Researcher level	Accommodating early career and emerging researchers	Yes: exclusively	4
		Yes: inclusive of other research levels	21
		No	1
		Unspecified	4
		Unclear	8
		NA	2
Traditional, complementary and integrative medicine sensitivity			
Paradigmatically-aligned	Alignment with core characteristics of the traditional, complementary and integrative medicine (e.g. consideration of the philosophical roots of the traditional, complementary and integrative medicine being studied)	Yes	9
		Scope allows	3
		Partial	1
		No	27
Intellectual property rights	Ethical approaches to intellectual property rights of traditional knowledge custodians	Yes	0
		Partial	6
		No	34
Tradition-informed framing	Tradition-informed communication and framing rather than biomedical framing	Yes	9
		Scope allows	3
		No	28
Person-centred	Person-centred translation (e.g. capacity for holistic, person-centred models and treatments to be measured; individualized; patient engagement)	Yes	1
		Scope allows	12
		No	27
Interpretation	Accuracy of interpretation (e.g. consideration of changes to interpretation of traditional, complementary and integrative medicine practices and treatments over time)	Yes	4
		Scope allows	6
		No	30
Stakeholder-informed transferability	Transferability of traditional knowledge considered (through participatory research or other forms stakeholder engagement)	Yes	2
		Scope allows	13
		No	25
Traditional, complementary and integrative medicine resource accessibility	Accessibility and integrity of traditional resources considered (e.g. are the necessary materials, equipment and facilities available?)	Yes	4
		Scope allows	5
		No	31
Needs assessment	Comparative benefit and need (health and disease landscape, patient preferences and values)	Yes	5
		Scope allows	7
		No	28
**Country level (*n *= 12*)***			
Research innovation			
Funder mechanism diversity^a^	Philanthropic, competitive government funding and block funding	Yes (three funding mechanisms)	1
		Partial (two funding mechanisms)	7
		No (one funding mechanism)	4
Diversity of funding type^a^	Diverse funding types such as individual, project and institutional	Yes (more than two types)	7
		Partial (two types)	1
		No (one only)	2
		NA	2
Diversity of funding size^a^	Diverse range of funding size per grant: mix across small (< US$ 100 000), mid (US$ 100 00–400 000) and large (> US$ 400 000) grants; small grants allow researchers to take risks	Yes (not limited, or across more than two funding ranges)	2
		Partial (across more than one funding range, or inclusive of small funding amounts)	8
		No (within one funding range, no small funding amounts)	1
Research capacity-building			
Diverse funding size^a^	Diverse range of funding size per grant: mix across small (< US$ 100 000), mid (US$ 100 000–400 000) and large (> US$ 400 000) grants; small grants allow researchers to build track record, larger grants provide opportunity to build programmes of research	Yes (not limited, or across more than two funding ranges),	2
		Partial (across more than one funding range, or inclusive of small funding amounts)	8
		No (within one funding range, no small funding amounts)	1
		NA	1

NA: not available.

^a^ Assessed at country level, hence sample size only 12.

## Results

We identified 39 927 grants listed in Dimensions, with the earliest grant recorded in 1960. Of these, 34 292 (86.5%) had data on duration, 27 019 (67.9%) reported funding data and 15 273 (38.3%) were categorized by research topic and health condition.

### Funding landscape

**Fig. 1:**
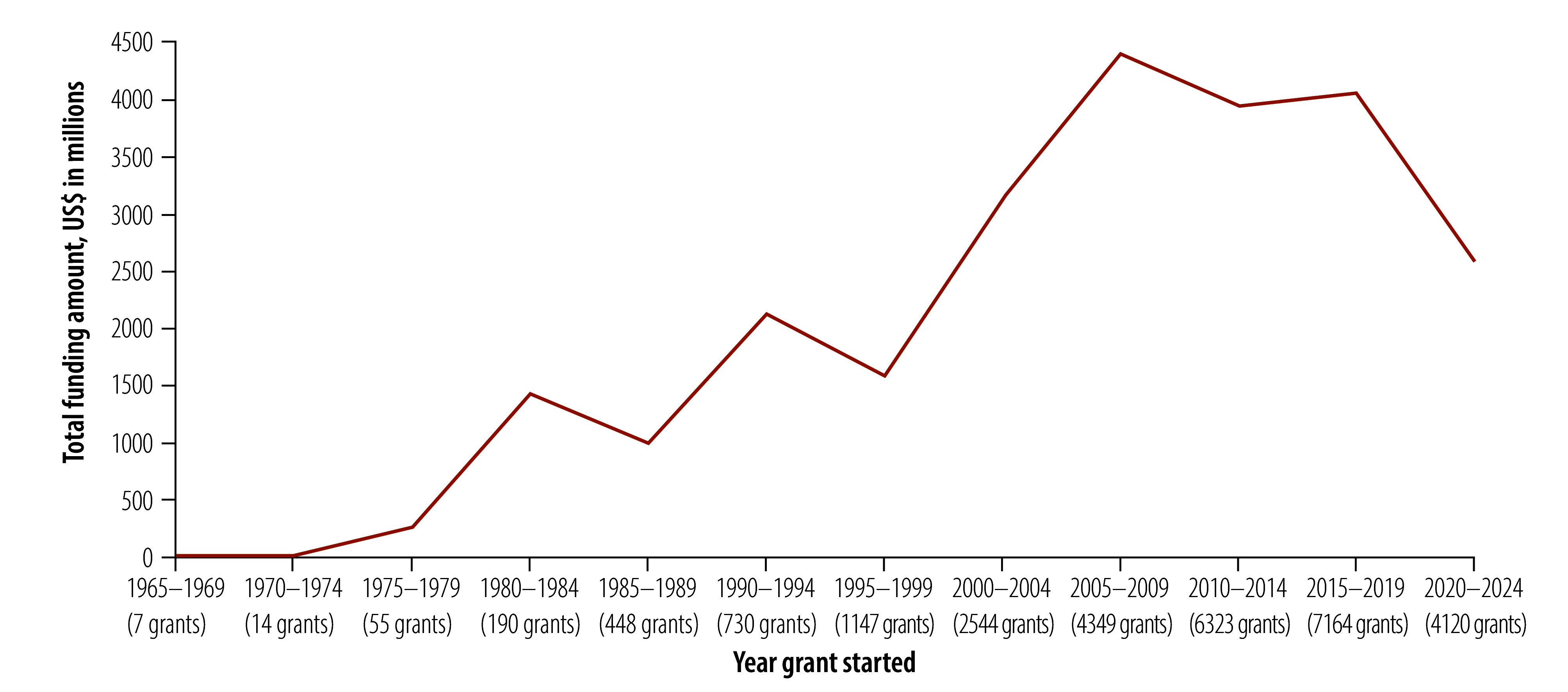
Total funding amounts for traditional, complementary and integrative medicine, 1960–2024

**Fig. 2:**
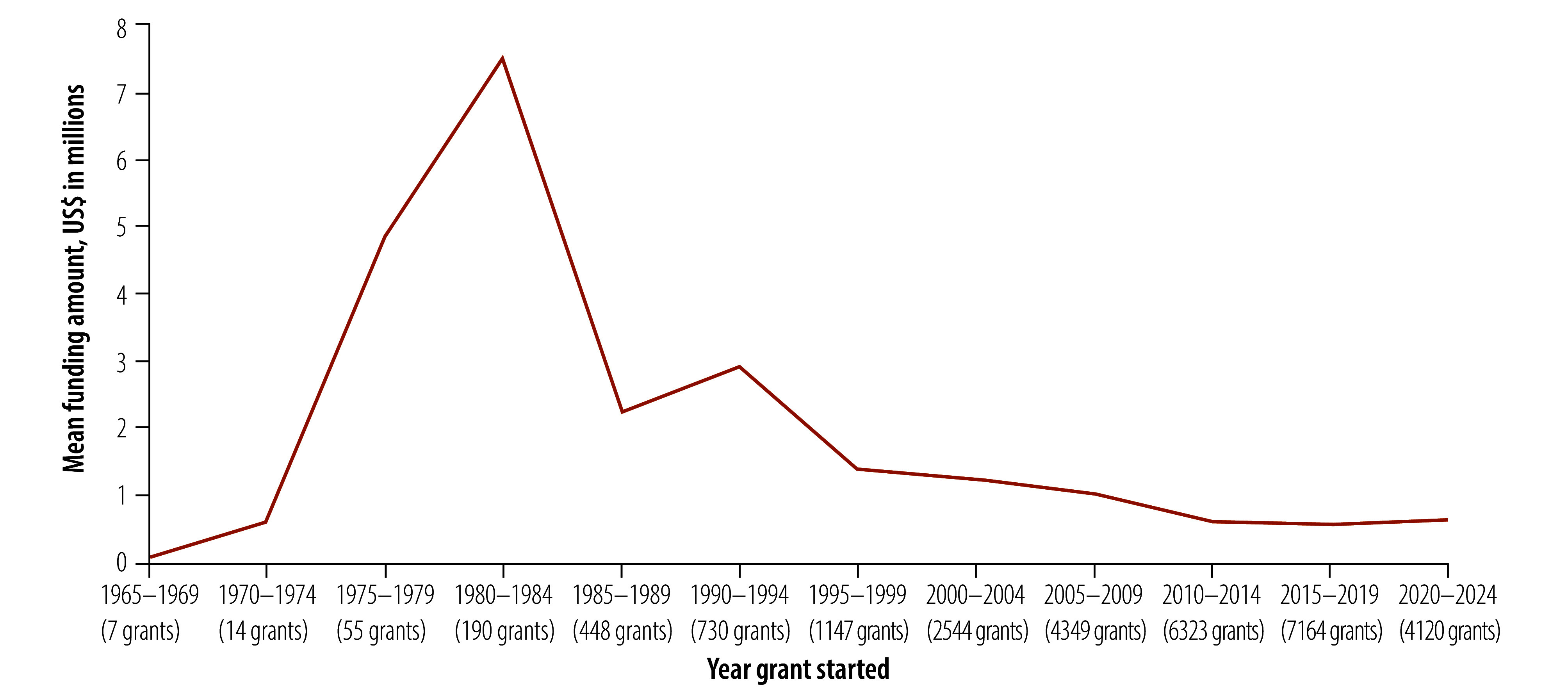
Mean funding amounts for traditional, complementary and integrative medicine, 1960–2024

**Fig. 3:**
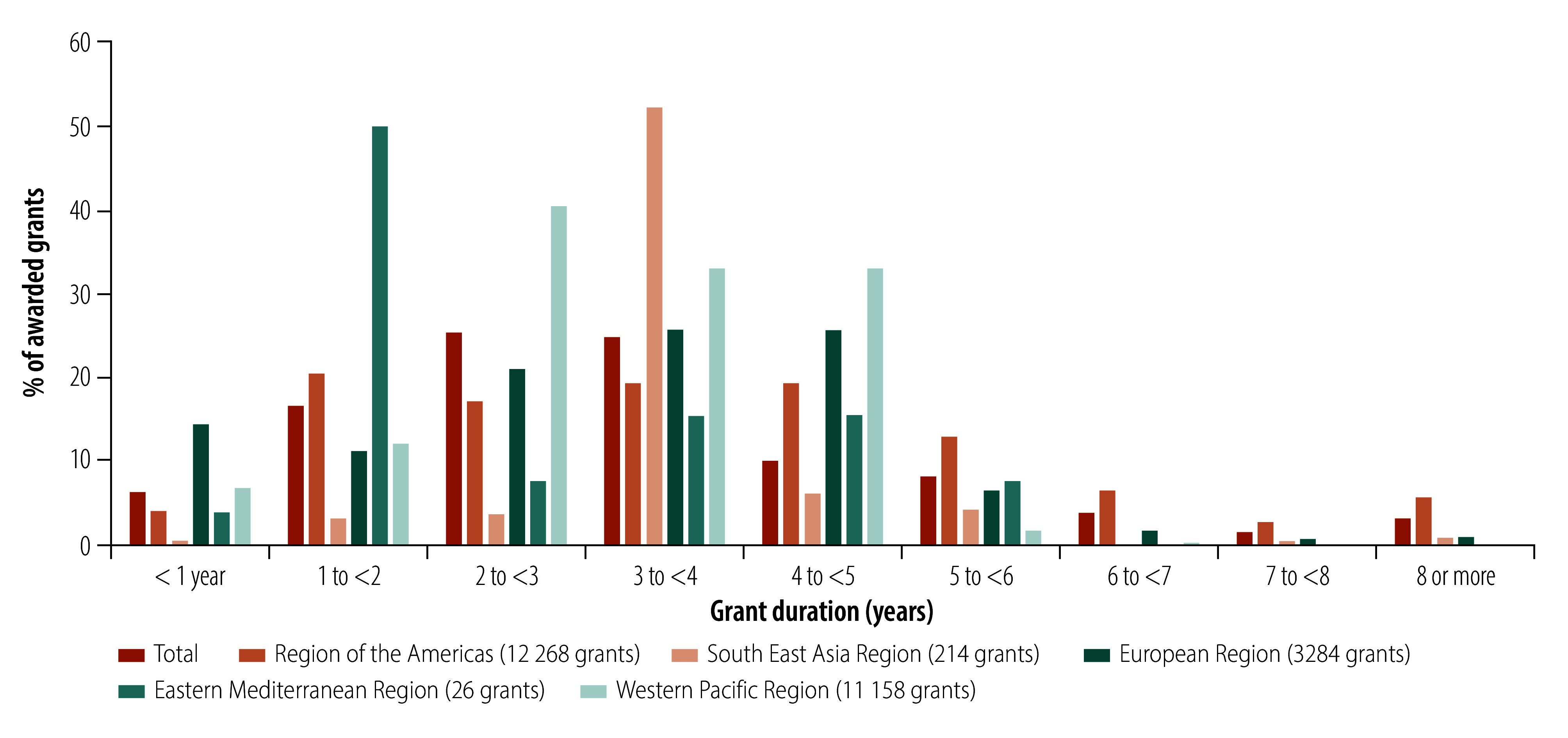
Duration of awarded grants for traditional, complementary and integrative medicine, by WHO region, 1960–2024

The 27 019 grants with recorded funding amounts were awarded between 1965 and 2025, with total funding amounting to US$ 24.5 billion (online repository).^14^ Of these, 42.6% (11 548 grants) were valued up to US$ 100 000, while 25.3% (6857 grants) ranged between US$ 100 000 and US$ 400 000. The highest total amount awarded was for the period between 2005 and 2010 (Fig. 1). While the number of indexed grants increased markedly until 2019, their mean value declined, and between 2020 and 2024, the mean value for the 4120 grants awarded was US$ 629 804 (Fig. 2). Half (50.3%; 17 173) of the grants with duration information had an award period of 2–4 years (Fig. 3).

WHO: World Health Organization.

Note: no funding amounts for grants from the African Region were reported in the Dimensions database. The absolute numbers are available in the online repository.^14^

Table 2 presents funding allocations for grants categorized by research topic and health condition. Of these, 10 812 grants (70.8%) had recorded funding amounts. About half of research grants were allocated to cancer (34.8%; 5318 grants) and cardiovascular diseases (20.1%; 3067 grants), with approximately US$ 5.8 billion and US$ 2.2 billion awarded, respectively. Mean grant values varied by health condition, with the lowest average funding awarded to research on skin conditions (mean: US$ 341 163; standard deviation, SD: 459 242), and the highest awarded to research on congenital conditions (mean: US$ 2.2 million; SD: 3.6 million), despite few grants in this latter category (0.1%; 17 grants).

**Table 2:** Indexed grants allocated to research, condition and disease categorization, by WHO region

Health condition category	No. (%) of grants^a^	Amount funded, US$ (*n* = 10 812)			WHO region, no. (%)						*P^b^*
		Total	Mean (SD)		African	Americas	South-East Asia	European	Eastern Mediterranean	Western Pacific	
Cancer	5318 (34.8)	5 764 254 232	1 470 473 (14 280 056)		86 (1.6)	3409 (64.6)	19 (0.4)	580 (11.0)	6 (0.1)	1174 (22.3)	< 0.001
Cardiovascular	3067 (20.1)	2 247 732 117	1 038 693 (3 088 213)		73 (2.4)	1778 (58.6)	12 (0.4)	405 (13.3)	3 (0.1)	764 (25.2)	< 0.001
Oral and gastrointestinal system	2107 (13.8)	1 144 581 469	849 096 (2 318 899)		22 (1.1)	1347 (64.8)	5 (0.2)	372 (17.9)	2 (0.1)	332 (16.0)	< 0.001
Infection	1629 (10.7)	896 476 966	908 285 (1 748 120)		140 (8.7)	986 (61.5)	11 (0.7)	287 (17.9)	0 (0.0)	180 (11.2)	< 0.001
Metabolic and endocrine system	1260 (8.2)	623 487 660	770 689 (2 318 899)		30 (2.4)	719 (57.8)	10 (0.8)	136 (10.9)	3 (0.2)	347 (27.9)	< 0.001
Musculoskeletal	1155 (7.6)	964 663 653	1 264 304 (3 588 746)		157 (13.7)	584 (50.8)	3 (0.3)	111 (9.7)	0 (0.0)	294 (25.6)	< 0.001
Inflammatory and immune system	1138 (7.5)	601 880 959	712 285 (1 628 304)		13 (1.2)	595 (52.6)	1 (0.1)	134 (1.9)	0 (0.0)	388 (34.3)	< 0.001
General relevance^c^	963 (6.3)	913 957 876	1 218 611 (2 984 216)		26 (2.7)	543 (56.9)	1 (0.1)	212 (22.2)	1 (0.1)	172 (18.0)	< 0.001
Respiratory	281 (1.8)	266 809 547	1 160 042 (2 688 271)		7 (2.5)	130 (46.9)	1 (0.4)	33 (11.9)	0 (0.0)	106 (38.3)	< 0.001
Stroke	133 (0.9)	58 629 690	553 110 (1 219 682)		3 (2.3)	57 (43.2)	1 (0.8)	7 (5.3)	0 (0.0)	64 (48.5)	< 0.001
Renal and urogenital system	88 (0.6)	46 369 677	747 898 (1 115 )		0 (0.0)	63 (71.6)	0 (0.0)	10 (11.4)	0 (0.0)	15 (17.0)	0.18
Skin	85 (0.6)	17 399 320	341 163 (459 242)		1 (1.2)	49 (58.3)	0 (0.0)	9 (10.7)	0 (0.0)	25 (29.8)	0.62
Injury and accident	74 (0.5)	53 792 170	1 014 947 (1 710 677)		10 (13.7)	40 (54.8)	0 (0.0)	9 (12.3)	0 (0.0)	14 (19.2)	0.001
Neurological	32 (0.2)	32 115 122	1 235 197 (1 467 216)		0 (0.0)	20 (64.5)	1 (3.2)	6 (19.4)	0 (0.0)	4 (12.9)	0.07
Disputed etiology	18 (0.1)	12 865 270	756 781 (1 429 293)		1 (5.6)	6 (33.3)	0 (0.0)	2 (11.1)	0 (0.0)	9 (50.0)	0.17
Ear	18 (0.1)	6 308 119	394 257 (611 686)		2 (11.1)	8 (44.4)	0 (0.0)	3 (16.7)	0 (0.0)	5 (27.8)	0.57
Congenital	17 (0.1)	21 925 925	2 192 593 (3 601 535)		1 (5.9)	11 (64.7)	0 (0.0)	4 (23.5)	0 (0.0)	1 (5.9)	0.53
Blood	17 (0.1)	11 447 687	763 179 (569 869)		0 (0.0)	9 (52.9)	1 (5.9)	5 (29.4)	0 (0.0)	2 (11.8)	0.003
**Total**	**15 273 (100.0)**	**13 684 697 459**	**1 158 235 (8 874 424)**		**551 (3.6)**	**8992 (59.4)**	**60 (0.4)**	**1954 (12.9)**	**13 (0.1)**	**3559 (23.5)**	**NA**

NA: not applicable; SD: standard deviation; US$: United States dollars.

^a^ Grants were able to be allocated to more than one category.

^b^ *P*-values denote differences between regions.

^c^ Includes grants that do not have a specific match to the existing categories.

Note: the categories maternal and reproductive health and mental health had no grants allocation and therefore not shown. Some inconsistencies arise in some values due to rounding.

Text analysis of all available grant abstracts (37 320) showed that treatment-related concepts such as vitamins (52.1%; 19 461), acupuncture (25.8%; 9633) and medicinal plants (11.7%; 4374; Table 3) were most common. Least frequently referenced were terms linked to systems of medicine, such as Unani (< 0.1%; 12), anthroposophy (< 0.1%; 14) and homeopathy (0.1%; 58); or other related research topics, such as ethnobotany (0.1%; 54), biodiversity (1.6%; 586) and digital health (1.1%; 425).

**Table 3:** Frequency of terms in abstracts of included grants for research on traditional, complementary and integrative medicine, 1960 to 2025

Term	No. (%)^a^ (*n* = 37 320)
Vitamin	19 461 (52.1)
Acupuncture	9633 (25.8)
Medicinal plants	4374 (11.7)
Yoga	2209 (5.9)
Meditation	1910 (5.1)
Massage	1279 (3.4)
Biodiversity	586 (1.6)
Indigenous	447 (1.2)
Digital health	425 (1.1)
Chiropractic	344 (0.9)
Cupping	143 (0.4)
Osteopathy	81 (0.2)
Naturopathy	71 (0.2)
Ayurveda	67 (0.2)
Homeopathy	58 (0.1)
Ethnobotany	54 (0.1)
Anthroposophy	14 (< 0.1)
Unani	12 (< 0.1)

^a^ Abstracts can contain more than one term, hence the total sum grants does not equal the sample size. Grants were able to be allocated to more than one category.

#### WHO regions

**Fig. 4:**
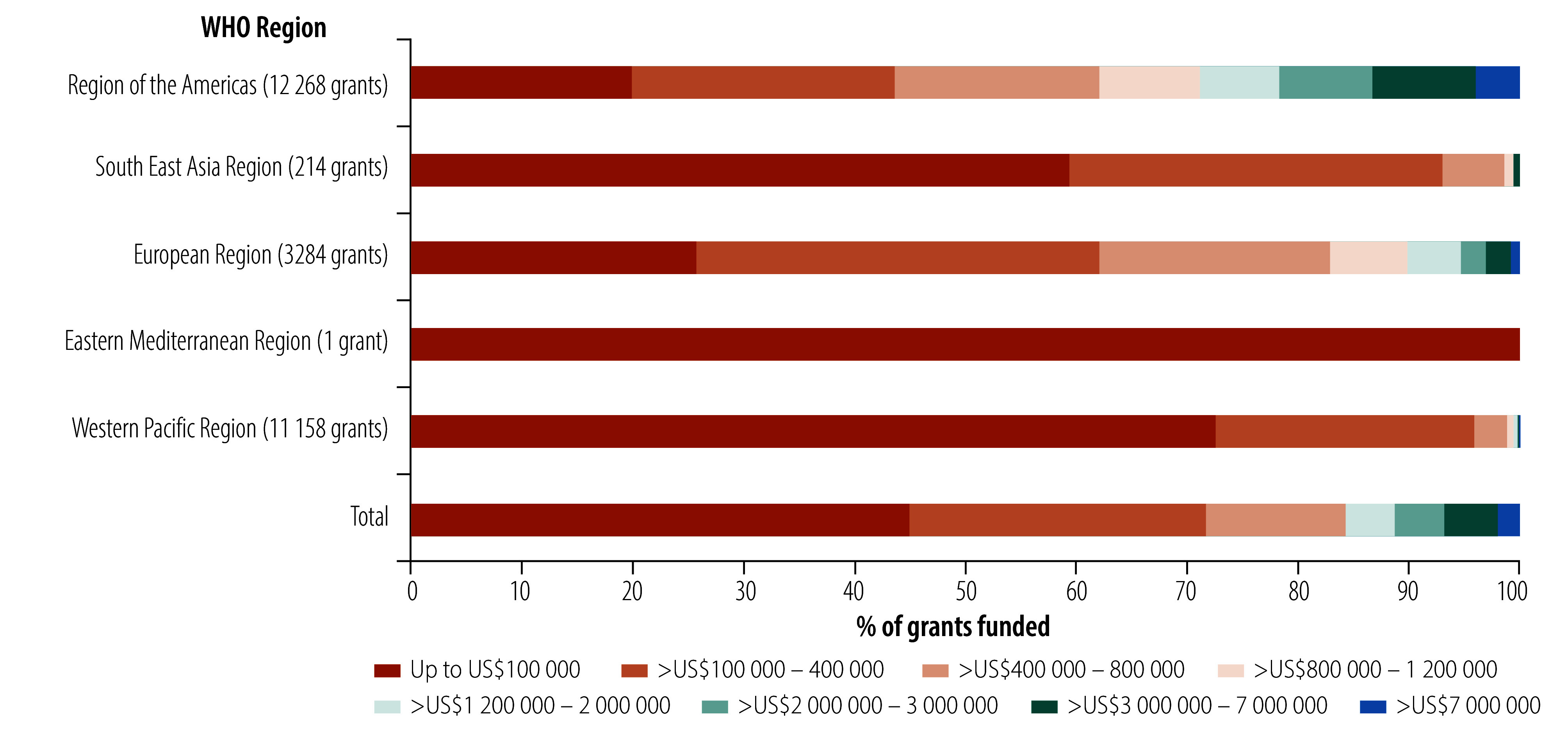
Grant distribution by funding size for traditional, complementary and integrative medicine, across WHO regions, 1960–2024

In assessing the countries where grants were awarded, we identified eight countries in the African Region, eight countries in the Region of the Americas, six in the South-East Asia Region, 29 in the European Region, five in the Eastern Mediterranean Region and nine in the Western Pacific Region. Of the 39 515 (99.2%) grants with recorded funder locations, funders were primarily located in the Region of the Americas (52.5%; 20 728), the Western Pacific Region (29.4%; 11 601) and the European Region (12.5%; 4937). The African Region (5%; 1978), the South-East Asia Region (0.6%; 244) and the Eastern Mediterranean Region (0.1%; 27) were less represented. There was a significant difference by WHO region with a moderate effect size (*P-*value: < 0.001) whereby, compared to other regions, the Region of the Americas had a greater proportion of grants funded for more than 5 years (27.8%; 4958/17 816) and the European Region had more grants awarded for less than 1 year (14.4%; 665/4618). No funding amounts were available in Dimensions for grants in the African Region; however there was a significant difference in grant amounts across other regions with a relatively strong effect size (*P*-value: < 0.001; Fig. 4). We identified funding from philanthropic organizations in the Region of the Americas, the European and the Western Pacific Regions.

US$: United States dollars; WHO: World Health Organization.

Note: no funding amounts for grants from the African Region were reported in the Dimensions database. The absolute numbers are available in the online repository.^14^

The proportion of funding awarded by region differed significantly across a range of health condition categories, although the effect size was found to be weak or negligible (Table 2). In the Eastern Mediterranean Region, the grant covered only four categories, whereas the Region of the Americas and the European Region had grants addressing each category. Notably, we did not identify funding for conditions within the categories of reproductive and maternal health or mental health in the Dimensions database.

#### Country level

We analysed funding across the 194 Member States (Table 4 available at https://www.who.int/publications/journals/bulletin/ and online repository).^14^ In the Dimensions database, there was evidence of active research funding in 66 (34.0%) countries and research grant funding in 45 countries (23.2%). Of the countries with research grant funding recorded in Dimensions, only seven (15.6%) countries had funding schemes explicitly including traditional medicine as a research topic, and 19 (42.2%) received grant funding from philanthropic sources.

**Table 4:** Traditional, complementary and integrative medicine infrastructure and funding by country, 2025

Country, by WHO region	Funding landscape for traditional, complementary and integrative medicine							
	Any research funding	Any grant funding identified	Specific grant funding	Philanthropic grant funding	National centre	National policy or framework	Government department, ministry or agency	Regulation of products or practice
**African Region**								
Benin	No	No	NA	NA	No	Yes	No	Yes
Burkina Faso	No	No	NA	NA	No	Yes	NA	NA
Burundi	No	No	NA	NA	No	Yes	Yes	Yes
Cameroon	Yes	No	NA	NA	Yes	Yes	Yes	Yes
Central African Republic	No	No	NA	NA	No	Yes	No	No
Chad	No	No	NA	NA	No	Yes	No	No
Comoros	No	No	NA	NA	No	Yes	No	No
Congo	No	No	NA	NA	No	Yes	Yes	No
Côte d'Ivoire	No	No	NA	NA	No	Yes	No	Yes
Democratic Republic of the Congo	No	No	NA	NA	No	Yes	No	Yes
Equatorial Guinea	No	No	NA	NA	No	Yes	Yes	Yes
Eritrea	No	No	NA	NA	No	Yes	No	Yes
Eswatini	No	No	NA	NA	NA	NA	NA	NA
Ethiopia	Yes	No	NA	NA	Yes	Yes	No	Yes
Gabon	Yes	No	NA	NA	Yes	Yes	Yes	No
Gambia	No	No	NA	NA	No	Yes	Yes	No
Ghana	Yes	No	NA	NA	Yes	Yes	No	Yes
Guinea-Bissau	No	No	NA	NA	No	Yes	Yes	Yes
Kenya	No	No	NA	NA	NA	NA	NA	NA
Liberia	No	No	NA	NA	No	Yes	No	Yes
Madagascar	No	No	NA	NA	No	Yes	Yes	Yes
Malawi	No	No	NA	NA	No	NA	NA	NA
Mali	No	No	NA	NA	NA	Yes	Yes	Yes
Mozambique	Yes	No	NA	NA	Yes	Yes	Yes	Yes
Namibia	No	No	NA	NA	No	Yes	No	No
Niger	No	No	NA	NA	No	Yes	Yes	Yes
Sao Tome and Principe	No	No	NA	NA	No	Yes	No	No
Senegal	No	No	NA	NA	No	Yes	Yes	Yes
South Africa	Yes	Yes	No	No	No	Yes	Yes	No
Togo	No	No	NA	NA	NA	NA	NA	NA
Uganda	Yes	No	NA	NA	Yes	Yes	No	Yes
United Republic of Tanzania	Yes	No	NA	NA	Yes	Yes	Yes	Yes
**Region of the Americas**								
Argentina	No	No	NA	NA	No	NA	No	Yes
Bahamas	Yes	Yes	No	Yes	No	NA	NA	NA
Barbados	Yes	Yes	Yes	No	NA	No	No	No
Belize	No	No	NA	NA	No	No	No	No
Bolivia (Plurinational State of)	No	No	NA	NA	No	Yes	Yes	Yes
Brazil	Yes	Yes	No	Yes	No	Yes	Yes	Yes
Canada	Yes	Yes	Yes	Yes	No	Yes	Yes	Yes
Chile	Yes	Yes	No	NA	No	Yes	Yes	Yes
Colombia	No	No	NA	NA	No	No	No	Yes
Costa Rica	No	No	NA	NA	No	No	No	Yes
Cuba	No	No	NA	NA	No	Yes	Yes	Yes
Ecuador	No	No	NA	NA	No	Yes	No	Yes
El Salvador	No	No	NA	NA	No	No	No	Yes
Grenada	No	No	NA	NA	No	No	Yes	Yes
Guatemala	No	No	NA	NA	No	No	No	No
Guyana	No	No	NA	NA	No	No	No	No
Haiti	No	No	NA	NA	No	Yes	Yes	Yes
Honduras	No	No	NA	NA	No	No	No	Yes
Mexico	No	No	NA	NA	No	Yes	Yes	Yes
Nicaragua	Yes	No	NA	NA	Yes	Yes	No	No
Panama	No	No	NA	NA	No	Yes	Yes	No
Paraguay	No	No	NA	NA	No	No	No	Yes
Peru	Yes	No	NA	NA	Yes	Yes	Yes	Yes
Saint Lucia	No	No	NA	NA	No	No	No	No
Saint Vincent and the Grenadines	No	No	NA	NA	No	No	No	No
Suriname	No	No	NA	NA	NA	NA	No	Yes
Trinidad and Tobago	No	No	NA	NA	No	No	Yes	No
United States	Yes	Yes	Yes	Yes	Yes	Yes	No	Yes
Uruguay	No	No	NA	NA	No	No	No	No
**South-East Asia Region**								
Bangladesh	No	No	NA	NA	No	Yes	Yes	Yes
Bhutan	Yes	No	NA	NA	Yes	Yes	Yes	Yes
Democratic People’s Republic of Korea	Yes	No	NA	NA	Yes	Yes	Yes	Yes
India	Yes	Yes	Yes	No	Yes	Yes	Yes	Yes
Maldives	No	No	NA	NA	No	Yes	Yes	Yes
Myanmar	No	No	NA	NA	No	Yes	Yes	Yes
Nepal	Yes	Yes	Yes	NA	Yes	Yes	Yes	Yes
Sri Lanka	Yes	No	NA	NA	Yes	Yes	Yes	Yes
Thailand	Yes	No	NA	NA	Yes	Yes	Yes	Yes
Timor-Leste	No	No	NA	NA	No	No	NA	NA
**European Region**								
Albania	No	No	NA	NA	No	No	No	Yes
Andorra	No	No	NA	NA	No	No	No	Yes
Armenia	No	No	NA	NA	No	No	No	Yes
Austria	Yes	Yes	No	Yes	No	No	Yes	Yes
Azerbaijan	No	No	NA	NA	No	No	No	Yes
Belarus	No	No	NA	NA	No	No	No	Yes
Belgium	Yes	Yes	No	Yes	No	No	Yes	Yes
Bosnia and Herzegovina	No	No	NA	NA	No	No	No	Yes
Bulgaria	No	No	NA	NA	NA	NA	No	Yes
Croatia	Yes	Yes	No	NA	No	No	No	Yes
Cyprus	No	No	NA	NA	No	Yes	No	Yes
Czechia	Yes	Yes	No	NA	No	No	No	Yes
Denmark	Yes	Yes	No	Yes	No	No	No	Yes
Estonia	Yes	Yes	No	NA	No	No	Yes	Yes
Finland	Yes	Yes	No	Yes	No	No	Yes	Yes
France	Yes	Yes	No	No	NA	NA	NA	NA
Germany	Yes	Yes	No	Yes	No	Yes	Yes	Yes
Hungary	Yes	Yes	No	NA	Yes	Yes	Yes	Yes
Iceland	Yes	Yes	No	NA	No	Yes	Yes	Yes
Ireland	Yes	Yes	No	No	No	No	Yes	NA
Israel	Yes	Yes	No	Yes	No	No	Yes	Yes
Italy	Yes	Yes	No	Yes	NA	NA	NA	NA
Latvia	Yes	Yes	No	NA	NA	NA	NA	NA
Lithuania	No	No	NA	NA	No	No	Yes	Yes
Luxembourg	Yes	Yes	No	NA	NA	NA	NA	NA
Malta	No	No	NA	NA	No	No	Yes	Yes
Montenegro	No	No	NA	NA	No	Yes	Yes	Yes
Netherlands (Kingdom of the)	Yes	Yes	No	Yes	No	No	Yes	Yes
Norway	Yes	Yes	No	Yes	Yes	No	Yes	Yes
Poland	Yes	Yes	No	Yes	No	Yes	Yes	Yes
Portugal	Yes	Yes	No	No	No	Yes	Yes	Yes
Republic of Moldova	No	No	NA	NA	No	No	No	Yes
Romania	No	No	NA	NA	No	No	No	Yes
Russian Federation	Yes	Yes	No	No	NA	NA	NA	NA
Serbia	No	No	NA	NA	No	Yes	Yes	Yes
Slovakia	Yes	Yes	No	No	No	Yes	Yes	Yes
Slovenia	Yes	Yes	No	NA	No	No	No	Yes
Spain	Yes	Yes	No	No	No	No	No	Yes
Sweden	Yes	Yes	No	Yes	No	Yes	No	Yes
Switzerland	Yes	Yes	No	Yes	No	Yes	No	Yes
Tajikistan	No	No	NA	NA	NA	NA	NA	NA
Türkiye	Yes	Yes	No	No	No	Yes	Yes	Yes
Ukraine	Yes	No	NA	NA	Yes	Yes	No	Yes
United Kingdom	Yes	Yes	Yes	Yes	No	Yes	Yes	Yes
**Eastern Mediterranean Region**								
Afghanistan	No	No	NA	NA	No	No	Yes	No
Bahrain	No	No	NA	NA	No	Yes	No	Yes
Iran (Islamic Republic of)	Yes	No	NA	NA	Yes	Yes	Yes	Yes
Iraq	No	No	NA	NA	No	Yes	No	Yes
Jordan	No	No	NA	NA	No	Yes	Yes	Yes
Kuwait	No	No	NA	NA	No	No	No	Yes
Lebanon	No	No	NA	NA	No	No	No	No
Morocco	No	No	NA	NA	No	No	No	Yes
Oman	No	No	NA	NA	No	Yes	Yes	Yes
Pakistan	Yes	No	NA	NA	Yes	Yes	Yes	Yes
Qatar	Yes	Yes	No	No	No	Yes	No	Yes
Saudi Arabia	Yes	No	NA	NA	Yes	Yes	No	Yes
Somalia	No	No	NA	NA	No	Yes	No	No
Sudan	Yes	No	NA	NA	Yes	Yes	No	Yes
Syrian Arab Republic	No	No	NA	NA	No	NA	Yes	Yes
Tunisia	No	No	NA	NA	No	Yes	No	Yes
United Arab Emirates	Yes	Yes	No	NA	Yes	Yes	Yes	Yes
Yemen	No	No	NA	NA	No	No	No	Yes
**Western Pacific Region**								
Australia	Yes	Yes	No	Yes	No	Yes	Yes	Yes
Brunei Darussalam	No	No	NA	NA	No	No	Yes	Yes
Cambodia	Yes	No	NA	NA	Yes	Yes	Yes	Yes
China	Yes	Yes	No	No	Yes	Yes	Yes	Yes
Cook Islands	No	No	NA	NA	No	No	No	No
Fiji	No	No	NA	NA	No	Yes	Yes	No
Indonesia	No	No	NA	NA	No	Yes	Yes	Yes
Japan	Yes	Yes	No	Yes	No	No	No	Yes
Kiribati	No	No	NA	NA	No	No	No	No
Lao People’s Democratic Republic	Yes	No	NA	NA	Yes	Yes	Yes	Yes
Malaysia	Yes	Yes	No	NA	NA	Yes	Yes	Yes
Marshall Islands	No	No	NA	NA	No	No	No	No
Micronesia (Federated States of)	No	No	NA	NA	No	No	No	No
Mongolia	Yes	No	NA	NA	Yes	Yes	Yes	Yes
Nauru	No	No	NA	NA	No	Yes	No	No
New Zealand	No	No	NA	NA	No	Yes	Yes	No
Niue	No	No	NA	NA		Yes	No	No
Palau	No	No	NA	NA	No	No	No	No
Papua New Guinea	No	No	NA	NA	No	Yes	Yes	Yes
Philippines	Yes	Yes	Yes	No	Yes	Yes	Yes	Yes
Republic of Korea	Yes	Yes	Yes	NA	Yes	Yes	Yes	Yes
Samoa	No	No	NA	NA	No	Yes	No	No
Singapore	No	No	NA	NA	No	Yes	Yes	Yes
Solomon Islands	No	No	NA	NA	No	Yes	No	No
Tonga	No	No	NA	NA	No	No	No	No
Tuvalu	No	No	NA	NA	No	No	No	Yes
Vanuatu	No	No	NA	NA	No	No	No	No
Viet Nam	No	No	NA	NA	No	Yes	Yes	Yes

NA: not applicable; WHO: World Health Organization.

Note: we did not identify data pertaining to funding for traditional, complementary and integrative medicine for Algeria, Angola, Antigua and Barbuda, Botswana, Cabo Verde, Djibouti, Dominica, Dominican Republic, Egypt, Eswatini, Georgia, Greece, Guinea, Jamaica, Kazakhstan, Kenya, Kyrgyzstan, Lesotho, Libya, Mauritania, Mauritius, Monaco, Nigeria, Republic of North Macedonia, Rwanda, Saint Kitts and Nevis, San Marino, Seychelles, Sierra Leone, South Sudan, Tajikistan, Togo, Turkmenistan, Uzbekistan, Venezuela (Bolivarian Republic of), Zambia and Zimbabwe.

The most active philanthropic or charity-based funders, as reported in the Dimensions database, were located in the United States (47 funders), the United Kingdom of Great Britain and Northern Ireland (39 funders) and Canada (10 funders). Across all identified philanthropic funders, traditional medicine-related grants accounted for an average of 1.9% of their total awarded grants as of 23 January 2025 (median: 0.7%; range: 0.0–47.8; online repository).^14^

### Country infrastructure

We identified 30 countries having a government-funded national traditional medicine centre or a centre focused on one aspect of traditional medicine, such as medicinal plants, typically supported through block funding rather than competitive grants. Only eight of these countries also had grant schemes that had funded research for traditional medicine, and India, Philippines, Republic of Korea and the United States offered competitive funding opportunities for such research. Except for Norway, countries with a government-funded centre had a national policy or framework. Gabon and Nicaragua were the only countries with a national centre that did not have a regulatory role. Twenty-two countries also had a government department, ministry or agency explicitly responsible for traditional medicine.

### Case study analysis

We identified up to five prominent funders in four of the six WHO regions (Table 5). For the African Region, the National Research Foundation in South Africa was the only funder identified. In the Eastern Mediterranean Region, of the two funders identified, the Qatar National Research Fund represented 96.3% (26/27) of all grants. Indian funders represented all five funders in the South-East Asia Region, while in the remaining regions the funders were spread across countries. The top five funders for the Western Pacific Region provided 94.0% (10 909/11 601) of all traditional medicine research grants in the region, whereas the top five funders in the Region of the Americas and the European Region provided 51.3% (10 626/20 728) and 30.2% (1490/4937) of grants, respectively. The median proportion of each funder’s total grants allocated to traditional medicine research was 1.0% (range: 0.1–82.0).

**Table 5:** Top funders of traditional, complementary and integrative medicine research indexed in the Dimensions database, by WHO region, 1976–2025

Funder, by WHO region^a^	Country	No. (%) of traditional, complementary and integrative medicine grants from funder	% of traditional, complementary and integrative medicine grants in region^b^
**African Region**		**1978 (NA)**	**100.0**
National Research Foundation (*n* = 275 743)	South Africa	1978 (0.7)	100.0
Total of top funders (*n* = 275 743)		1978 (0.7)	100.0
**Region of the Americas**		**20 728 (NA) **	**100.0**
São Paulo Research Foundation (*n* = 224 236)	Brazil	3097 (1.4)	14.9
Coordenação de Aperfeiçoamento de Pessoal de Nível Superior (*n* = 205 203)	Brazil	2758 (1.3)	13.3
National Center for Complementary and Alternative Medicine (*n* = 2483)	United States	2035 (82.0)	9.8
National Cancer Institute (*n* = 73 859)	United States	1581 (2.1)	7.6
National Institute of Food and Agriculture (*n* = 72 373)	United States	1155 (1.6)	5.6
Total of top funders (*n* = 578 154)		10 626 (1.8)	51.3
**South East Asia Region**		**244 (NA)**	**100.0**
Department of Biotechnology (*n* = 7803)	India	136 (1.7)	55.7
Science and Engineering Board (*n* = 8416)	India	64 (0.8)	26.2
Indian Council of Medical Research (*n* = 760)	India	30 (3.9)	12.3
Department of Science and Technology (*n* = 1726)	India	7 (0.4)	2.9
DBT/Wellcome Trust India Alliance (*n* = 543)	India	7 (1.3)	2.9
Total of top funders (*n* = 19 248)		244 (1.3)	100.0
**European Region**		**4937 (NA)**	**100.0**
Russian Foundation for Basic Research (*n* = 440 636)	Russian Federation	393 (0.1)	8.0
National Science Center (*n* = 29 834)	Poland	346 (1.2)	7.0
Deutsche Forschungsgemeinschaft (*n* = 151 671)	Germany	254 (0.2)	5.1
Belgian Federal Science Policy Office (*n* = 51 655)	Belgium	253 (0.5)	5.1
European Commission (*n* = 155 198)	European Union Member States	244 (0.2)	4.9
Total of top funders (*n* = 828 994)		1490 (0.2)	30.2
**Eastern Mediterranean Region**		**27 (NA)**	**100.0**
Qatar National Research Fund (*n* = 4022)	Qatar	26 (0.6)	96.3
University of Sharjah (*n* = 481)	United Arab Emirates	1 (0.2)	3.7
Total of top funders (*n* = 4503)		27 (0.6)	100.0
**Western Pacific Region**		**11 601 (NA)**	**100.0**
National Natural Science Foundation of China (*n* = 561 771)	China	5798 (1.0)	50.0
Japan Society for the Promotion of Science (*n* = 1 032 253)	Japan	4346 (0.4)	37.5
Ministry of Health Labour and Welfare (*n* = 30 086)	Japan	347 (1.2)	3.0
National Health and Medical Research Council (*n* = 31 896)	Australia	246 (0.8)	2.1
Ministry of Higher Education (*n* = 5483)	Malaysia	182 (3.30)	1.6
Total of top funders (*n* = 1 661 489)		10 919 (0.7)	94.2

NA: not applicable; WHO: World Health Organization.

^a^ The sample sizes are the total number of grants the funders have awarded.

^b^ Denominators are the number of grants for each region.

#### Funder characteristics

We identified case study funders from twelve countries: Barbados, Brazil, Canada, China, India, the Philippines, Qatar, Republic of Korea, the Russian Federation, South Africa, the United Kingdom of Great Britain and Northern Ireland and the United States (online repository).^14^ These case studies included four funders identified through online searches for specific schemes, and the top grant funders in eight countries as listed in the Dimensions database. Six of these were chosen as the leading funder in their region and the remaining two were the leading funder in a country with one of the four identified grant schemes. Across these countries, 40 grant schemes were examined. Of these, 13 (35.1%) explicitly indicated traditional medicine as a focus. Among the 13 schemes that specified methodological or design directives, clinical trials (seven) were the most frequently referenced. Five schemes encouraged trials, three exclusively permitted them and two explicitly prohibited them. One scheme prioritized safety and efficacy without specifying a trial method. 

Funding data were available for eight of the Dimensions-indexed case study funders. These funders accounted for between 3.8% and 100.0% of traditional medicine research funding in their region, and between 20.3% and 100.0% in their respective countries (online repository).^14^ Five of the eight had awarded research grants before 2001. 

Between 11.7% (5170/44 151) of grant-associated publications from China and 86.9% (1194/1374) from the United Kingdom were indexed on Altmetrics, with citations spanning all metric categories. Policy documents most frequently cited outputs from grants awarded in countries where English is a prominent language.

#### Funding scheme critical appraisal

Funding scheme appraisal is summarized in Table 1 and detailed in the online repository.^14^ Based on criteria for supporting innovation and ground-breaking research, Brazil, China, India, the United Kingdom and the United States had funding landscapes that met all criteria either partially or fully. Across the 40 schemes analysed, flexibility was the most commonly met scheme-level criterion (30 schemes), while longer grant duration was the most frequently fully met criterion (16 schemes). At the country level, diversity in funding type emerged as the most common indicator of innovation, fully met by seven of the 12 countries included in the analysis.

Only schemes from the United States met all criteria for capacity-building support, while those from Canada and India partially met the criteria. The most-met capacity-building criterion was support for researchers, with 27 of 40 schemes including provisions for students or early-career researchers. In contrast, only six schemes fully met the criteria for including clinicians in the research process or ensuring clinical relevance.

Only the National Natural Science Foundation Key Programme in China met all criteria for sensitivity to paradigmatic differences and knowledge types. A further eight schemes met seven of the eight criteria, with none fully addressing intellectual property rights of knowledge custodians. Notably, only four of these nine schemes were among the 11 identified as being specific to traditional medicine.

## Discussion

Findings from this analysis of global research funding for traditional medicine suggest ways to strengthen the funding landscape (Box 1). Over the last 15 years, funding has declined, with traditional medicine receiving only 1.0% of global health research funding. This disparity undermines the strategic objective 1 of the WHO* Global traditional medicine strategy 2025–2034*,^11^ which aims to establish an evidence base for traditional medicine, and highlights the need for dedicated research funding through existing or new mechanisms. The lack of specific funding schemes often forces traditional medicine researchers to compete for mainstream schemes, where review panels may lack relevant expertise or hold ideological biases.^20^^,^^21^ This challenge is compounded by the marginalized sociocultural position of traditional medicine within existing health and economic power structures.^20^ Therefore, efforts to improve funding must also address marginalization, for example, by including traditional medicine experts on grant review panels and in decision-making bodies that influence research funding.

Box 1 **Analysis-informed suggestions to strengthen the global research funding landscape for traditional, complementary and integrative medicine**

- Establish dedicated funding for traditional, complementary and integrative medicine research via existing or new grant schemes.
- Include traditional, complementary and integrative medicine experts on grant assessment panels.
- Support both block and competitive traditional, complementary and integrative medicine research funding.
- Improve indexing of traditional, complementary and integrative medicine grants in global databases, such as Dimensions, especially for underrepresented regions and smaller schemes.
- Develop a specific grant classification system for traditional, complementary and integrative medicine.
- Create guidelines for funders to design grants aligned with the unique paradigms and methods of traditional, complementary and integrative medicine.

We found that many countries lack the focused policy, expertise, education and research support for traditional medicine typically provided by a government-funded national research centre.^22^ The analysis also revealed limited diversity in funding mechanisms or funder type in some countries. National centres often rely on stable block funding, while university-led research typically depends on competitive grants drawing on academic expertise.^22^ A combination of both approaches could build a more sustainable and innovative traditional medicine research ecosystem. However, block funding may be allocated to centres with broader health mandates. Future research should investigate the level and type of traditional medicine research within these general block-funded centres.

Our analysis revealed considerable data gaps in the global database, underscoring persistent challenges in estimating research funding for traditional medicine. Some funding schemes were not indexed in Dimensions, and many indexed grants lacked key details such as funding amounts. Furthermore, no data were available for several countries in the African Region and the Region of the Americas, despite high reported use of traditional medicine in these regions.^23^^,^^24^ Grant categorization within Dimensions also lacks granularity, which is problematic given the diversity of traditional medicine systems, treatments and interventions. A global classification system would support funding agencies, enhance database functionality, and enable more targeted searches. While Direction 2.1 of the WHO* Global traditional medicine strategy 2025–2034*^11^ advocates for standardized classifications of products within regulatory frameworks, our findings suggest this approach should be expanded to encompass research funding systems. Encouraging funders to share their data for indexing in global databases such as Dimensions would also support more complete and accurate future analyses.

Our case study analysis also revealed that major grant schemes often fail to accommodate paradigms and methods of traditional medicine.^25^^,^^26^ As a result, research is frequently forced to conform to biomedical research frameworks, which does not align with the traditional medicine strategy’s calls for better incorporation of traditional medicine concepts in policies and action plans (Direction 4.1) and more inclusive models for knowledge use (Direction 4.2).^11^ While these priorities are not specific to research, guidelines for designing funding schemes that accommodate the unique needs of traditional medicine research are essential to fully realize its potential for global health.

This study has several limitations. First, while our triangulated search strategy was designed to be comprehensive, it was not exhaustive; some grant schemes may have been missed, particularly those with limited indexing or online visibility. This issue is further compounded by limitations within the Dimensions database, including incomplete coverage of countries, restricted indexing sources and variable details from funders. Second, case study funders did not represent the majority of funders in the European and Western Pacific Regions, limiting generalizability. Third, poor performance against appraisal criteria may reflect the fact that some schemes were established before relevant frameworks were published, though this temporal mismatch is of minor importance, given the appraisal’s forward-looking purpose. Fourth, our classification of topics relied on the occurrence of traditional medicine-related terms in grant abstracts, which may have led to misclassification. To mitigate this risk, we included only abstracts already classified as traditional medicine within Dimensions. Finally, in the absence of a prior data set for comparison, our findings should be interpreted with caution regarding potential over- or under-estimation.

To conclude, current features of the global research funding landscape constrain the growth of traditional medicine and hinder the development of robust evidence. Limitations in the Dimensions database also restrict the ability to assess research trends, which is essential for effective coordination and planning. Transforming funding for traditional medicine research requires focused attention to ensure adequate and proportionate resourcing. Funders, in collaboration with key stakeholders, play an essential role in advancing traditional medicine research and supporting the vision of the WHO global traditional medicine strategy.
